# Optimizing the location of vaccination sites to stop a zoonotic epidemic

**DOI:** 10.1038/s41598-024-66674-x

**Published:** 2024-07-10

**Authors:** Ricardo Castillo-Neyra, Sherrie Xie, Brinkley Raynor Bellotti, Elvis W. Diaz, Aris Saxena, Amparo M. Toledo, Gian Franco Condori-Luna, Maria Rieders, Bhaswar B. Bhattacharya, Michael Z. Levy

**Affiliations:** 1 Department of Biostatistics, Epidemiology and Informatics, Perelman School of Medicine, University of Pennsylvania, Philadelphia, PA USA; 2 Zoonotic Disease Research Lab, One Health Unit, School of Public Health and Administration, Universidad Peruana Cayetano Heredia, Lima, Peru; 3 The Wharton School, University of Pennsylvania, Philadelphia, PA USA

**Keywords:** Viral infection, Infectious diseases, Diseases, Mathematics and computing, Applied mathematics, Scientific data, Statistics

## Abstract

Mass vaccinations are crucial public health interventions for curbing infectious diseases. Canine rabies control relies on mass dog vaccination campaigns (MDVCs) that are held annually across the globe. Dog owners must bring their pets to fixed vaccination sites, but sometimes target coverage is not achieved due to low participation. Travel distance to vaccination sites is an important barrier to participation. We aimed to increase MDVC participation in silico by optimally placing fixed-point vaccination locations. We quantified participation probability based on walking distance to the nearest vaccination site using regression models fit to participation data collected over 4 years. We used computational recursive interchange techniques to optimally place fixed-point vaccination sites and compared predicted participation with these optimally placed vaccination sites to actual locations used in previous campaigns. Algorithms that minimized average walking distance or maximized expected participation provided the best solutions. Optimal vaccination placement is expected to increase participation by 7% and improve spatial evenness of coverage, resulting in fewer under-vaccinated pockets. However, unevenness in workload across sites remained. Our data-driven algorithm optimally places limited resources to increase overall vaccination participation and equity. Field evaluations are essential to assess effectiveness and evaluate potentially longer waiting queues resulting from increased participation.

## Introduction

Zoonotic epidemics and pandemics are an increasing public health threat worldwide. In Latin America, Asia, and Africa, epidemics of rabies and other zoonotic diseases are ongoing in major urban centers^1–8^. Vaccination efforts to eliminate canine rabies from Latin American countries have been mostly successful^6^. However, Peru is experiencing the first instance of canine rabies reintroduction into an area previously declared free of transmission in Latin America^9^. In the city of Arequipa and surrounding provinces, continued and increased transmission in free-roaming dogs^10,11^, the sole animal reservoir in the region, has put more than a million human inhabitants at risk of rabies, a fatal, but entirely preventable, disease^12^. Annual mass dog vaccination campaigns (MDVCs) have been implemented in Peru to eliminate the epidemic without success^13^. The Pan American Health Organization recommends an annual canine mass vaccination coverage of 80%^14^; however, in Arequipa, this goal has not been attained in the past 8 years of vaccination campaigns, and rabies virus persists in the free-roaming dog population^11,13,15^.

Most MDVCs in Latin America and Africa rely on fixed-location vaccination posts, where vaccinators wait for dog owners to bring their dogs to a set place^15–19^. The extensive application of fixed-location vaccination is due to its relative ease of implementation and lower cost compared to other strategies^18,20,21^. However, in some contexts, fixed-point MDVCs have failed to attain coverage targets^13,22,23^. Extensive behavioral research has been conducted to reduce refusal of human vaccines^24–29^; recent observational studies have focused on understanding non-participation in MDVCs^13,15,16,18,19,30,31^. Among the barriers reported by dog owners to their participation in MDVCs are inconvenient locations and distance to the vaccination posts^15,16,18^. In Arequipa, Peru, participation in MDVCs directly decreases with each city block of distance from the dog owner’s household^13^. Because the rabies virus is transmitted at very low levels within dog populations^32^, the virus can persist within pockets of unvaccinated dogs^33–36^, making the prospects of elimination more difficult when MDVCs do not generate spatial evenness of protection^37^. Despite these reports, the locations of fixed vaccination posts are mainly determined by convenience and recognizability (e.g. the entrance to a health post, a well-known park)^13^. To address this problem, we present an algorithm that provides a data-driven strategy to guide the placement of fixed-point vaccination sites.

The algorithms we present here fall within the purview of ‘facility location problems’, which are a class of spatial optimization problems based on the assumption that the effectiveness of a facility is determined by some function of the distance traveled by those who visit it^38^. With increasing travel distance, facility accessibility decreases, and thus the location's effectiveness decreases^38,39^. This relationship holds for facilities such as libraries and schools, to which proximity is desirable^40^, and, based on previous field studies of canine rabies vaccination^13,15,18^, could also hold for MDVCs. Current-practice MDVC locations fail not only to achieve appropriate overall vaccination coverage, but also to improve vaccine equity by generating spatial evenness in vaccination coverage^13^.

The overarching aim of this study was to explore optimization procedures to increase fixed-point MDVC coverage. Using a spatial approach to improve the effectiveness of vaccination sites could not only increase overall vaccination coverage but may also create a more even geographic distribution of vaccination coverage. Our objective was to develop an optimization algorithm to determine the placement of vaccination points that would maximize vaccination coverage. We also evaluated vaccination spatial evenness, a dimension of vaccine equity, and the distribution of workload at vaccination sites. We present a comparison between our algorithm and results from a current-practice MDVC implemented in Arequipa, Peru, in an attempt to quell the dog rabies epidemic in the city.

## Methods

### Ethical statement

Ethical approval was obtained from Universidad Peruana Cayetano Heredia (approval number: 65369), Tulane University (approval number: 14–606720), and the University of Pennsylvania (approval number: 823736). All human subjects in this study were adults and informed consent was obtained from all subjects and/or their legal guardian(s). All methods were carried out in accordance with relevant guidelines and regulations.

### Data

Our data were collected from the Alto Selva Alegre (ASA) district in Arequipa City. The Ministry of Health (MOH) organizes an MDVC to vaccinate dogs against the rabies virus every year. Briefly, the MOH set up and staffed various fixed-location vaccination posts across the district. Vaccination tent locations were selected based on the convenience and intuition of the public health personnel^41^. A full description of MDVC operations in Arequipa is available elsewhere^13^. The data we used for our study consists of two sources: (1) geographic locations of the fixed-location vaccination posts and (2) georeferenced surveys conducted yearly in ASA immediately following the 2016–2019 MDVCs to ascertain household participation in the campaigns.

#### Vaccination post locations

**Figure 1:**
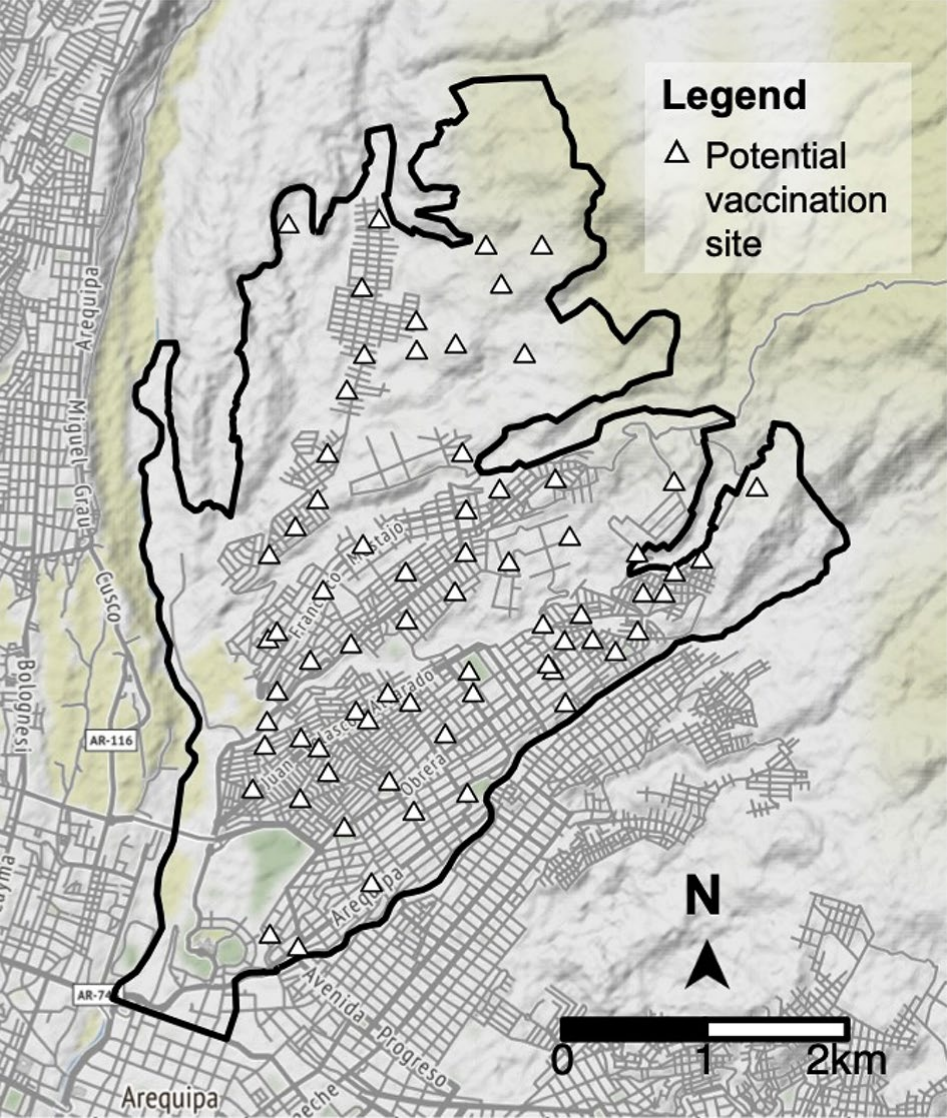
Alto Selva Alegre district with possible vaccination locations. The potential sites of fixed-location vaccination tents are depicted by white triangles. The optimization algorithms select the optimal locations among these possibilities. The map was created using R package *ggmap* version 4.0.0 (https://cran.r-project.org/web/packages/ggmap/).

Fixed-location vaccination posts used between 2016 and 2019 were georeferenced during the yearly MDVC by our team. In addition, all open sports fields, squares, parks, and other open spaces were georeferenced and then approved by MOH health inspectors to ensure they could be used as vaccination sites. Out of 85 potential sites, 15 were rejected by MOH officials as infeasible, leaving 70 potential sites for our optimization analysis (Fig. 1).

#### Vaccination campaign household surveys

Surveys following the annual MDVC were conducted from 2016 to 2019. Variables collected in these surveys and analyzed in this study include the geographic location of each house in the study area, household participation in the MDVC, number of dogs owned by the household, and number of dogs vaccinated in the MDVC. Data from all 4 years were used to construct regression models. The actual placement of vaccination tents in 2016 was used to demonstrate the benefits of optimizing the placement of vaccination sites. A full description of survey methods is available from Castillo-Neyra et al.^13^.

### Regression construction of participation probability

Distance to a fixed-location vaccination point is an important factor influencing a dog owner’s participation in an MDVC^13,15^. To further explore this relationship, we constructed a model to estimate MDVC participation probability as a function of the distance between each house and its closest MDVC site. Given that the vast majority of people bring their dogs on foot to the MDVC (about 95% according to the 2016 survey data), we calculated the shortest walking distance between each household in the study and the closest fixed-location vaccination point. The shortest walking distances were obtained using Mapbox Directions API and the Leaflet Routing Machine^42,43^. Information about household participation in each year’s vaccination campaign was obtained from 2016 to 2019 post-MDVC survey data.

We considered eight regression models to assess the relationship between participation probability and walking distance to the nearest vaccination point: (1) Poisson, (2) negative binomial, (3) binomial with linear distance terms, (4) binomial with linear and quadratic distance terms, and (5–8) mixed-effects versions of models 1–4 that incorporate a random-effect coefficient for year. For the Poisson and negative binomial models, we constructed 30 m distance bins and predicted the number of participating houses offset by the number of houses per bin. All models were evaluated using root mean square error (RMSE) and prediction error determined via fivefold cross-validation, as well as Aikake information criteria (AIC) (Table 1). The Poisson and negative binomial regression models were also compared to each other using the likelihood ratio test^44^.

**Table 1:** Regression analysis and cross-validation results.

Name	GLM regression equation	AIC	Prediction error, %	RMSE
Poisson	$log(\lambda_{x} )_{{}} = log\left( {n_{i} } \right) + \beta_{0} + \beta_{1} x$	455.48	− 0.11419503	0.6615610
Negative binomial	$log(\lambda_{x} )_{{}} = log\left( {n_{i} } \right) + \beta_{0} + \beta_{1} x$	457.48	− 0.11397836	0.6615612
Binomial—linear terms	$logit\left( {p_{x} } \right) = \beta_{0} + \beta_{1} x$	2837.4	− 0.11098002	2.4429062
Binomial—linear and quadratic terms	$logit\left( {p_{x} } \right) = \beta_{0} + \beta_{1} x + \beta_{2} x^{2}$	2457	− 0.11279694	2.4659023
Poisson mixed effects	$log(\lambda_{x} )_{{}} = log\left( {n_{i} } \right) + \beta_{0} + \beta_{1} x + \mu Z$	449.4	− 0.05125864	0.6309559
Negative binomial mixed effects	$log(\lambda_{x} )_{{}} = log\left( {n_{i} } \right) + \beta_{0} + \beta_{1} x + \mu Z$	451.4	− 0.05151737	0.6309566
Binomial—linear mixed effects	$logit\left( {p_{x} } \right) = \beta_{0} + \beta_{1} x + \mu Z$	2431.4	− 0.09721891	2.4506587
Binomial—linear and quadratic mixed effects	$logit\left( {p_{x} } \right) = \beta_{0} + \beta_{1} x + \beta_{2} x^{2} + \mu Z$	2433.2	− 0.09118541	2.4457406

*λ* refers to the number of expected houses participating per 30 m bin from the vaccination point, *n*_*i*_ refers to the number of houses per bin, *x* represents the distance (m) from the nearest vaccination tent (for binned data, mean distance per bin),* p* represents the probability of vaccination (per household), *Z* (in the mixed models) represents the year (2016–2019). Note AIC values were used only to compare fixed-effects vs. mixed-effects versions of a given model type (e.g., fixed-effects Poisson vs. mixed-effects Poisson).

### Optimal placement of tents

We simulated campaigns where placement of the vaccination tents was optimized using variants of the facility location problem. The goal in a facility location problem is to determine the placement of facilities (i.e., vaccination sites), among a pool of candidate sites, such that facility access for a set of demand points (i.e., houses) is optimized. Towards this, we first implemented the Teitz and Bart’s algorithm for the “*p*-median problem,” which aims to find a subset of vaccination sites, *S*, of size *p* among a set of candidates, where the average distance of all houses to their nearest point in *S* is minimized^44^. To apply this to mass dog vaccination in Arequipa, we aimed to find a set of 20 optimized tent locations that minimized average walking distance to the nearest vaccination site; the number of tents (*p* = 20) was selected to match the number of tents annually run by the MOH in the MDVC. To find the optimized sites we then applied the Teitz and Bart algorithm. To describe the algorithm denote the set of all houses by $H = \left\{ {h_{1} , h_{2} , \ldots , h_{N} } \right\}$, where *N* is the total number of houses. The Teitz and Bart algorithm for placing 20 vaccination sites proceeds as follows:

1. Select a random subset of 20 vaccination sites $S = \left\{ {s_{1} , s_{2} , \ldots , s_{20} } \right\}$, out of all candidate sites *A*. For each house $h_{i} \in H$, calculate $d_{min} \left( i \right)$, the walking distance of $h_{i}$ to the nearest site in *S*. Then calculate the average walking distance for all houses in the study area:
  $$\overline{{d_{min} }} = \frac{1}{N}\mathop \sum \limits_{i = 1}^{N} d_{min} \left( i \right)$$
2. Exchange $s_{1}$ with each candidate site in $A\backslash S$ and keep the one that minimizes $\overline{{d_{min} }}$.
3. Repeat step 2 with the remaining 19 sites in *S* until the objective function stabilizes.

The second optimization algorithm is an extension of the classic “*p*-center problem,” which aims to find the subset of facility sites that minimizes the maximum walking distance between any house and its nearest facility^45^. Classically, the *p*-center problem is formulated using Euclidean distance, and the solution involves drawing *p* circles whose union encloses all the demand points such that the maximum of the radii of the circles is minimized^45^. However, the classic formulation of the *p*-center problem yields many redundant solutions for our case, which uses walking distance instead of Euclidean distance. As a result, we adapted the *p-*center problem to minimize the sum of the 10 *largest maximal tent distances*, which we define as follows. For each vaccination tent $s \in S$, let $C_{s}$ represent all houses in *H* for which the tent *s* is its closest facility. Let $D_{s} = \left\{ {d_{min} \left( i \right):i \in C_{s} } \right\}$ be the set of walking distances between the tent *s* and all houses in its catchment. Denote by

$$max\left( {D_{s} } \right) = max_{{i \in C_{s} }} d_{min} \left( i \right),$$

the maximal distance from the tent *s* to the houses in its catchment. Now, denote by

$$\left\{ {m_{1} ,m_{2} , \ldots ,m_{10} } \right\}$$

the 10 maximal values among $\left\{ {max\left( {D_{1} } \right), max\left( {D_{2} } \right), \ldots ,max\left( {D_{20} } \right)} \right\}.$ Our *p-*center algorithm is similar to the *p*-median algorithm outlined above; where, instead of minimizing the average walking distance $\overline{{d_{min} }}$, our *p*-center algorithm minimizes $\mathop \sum \limits_{i = 1}^{10} m_{i}$.

In addition, we devised a third optimization algorithm that utilized our best-fit regression model that estimated MDVC participation probability as a function of the walking distance between each house and its nearest vaccination site. We used this regression function to calculate the expected probability that a household would participate in an MDVC based on the shortest distance its occupants would have to walk to arrive at a vaccination site for a selected set of candidate sites. These probabilities can be averaged among all households to obtain the expected vaccination coverage achieved for a particular set of MDVC sites. For this final algorithm that we term *p-probability*, we applied a similar recursive interchange technique as the *p*-median and *p*-center algorithms outlined above, except we maximized expected MDVC participation as our objective function rather than minimizing walking distance.

We provided the algorithms with the georeferenced locations of all houses and candidate vaccination sites in the study area, along with a matrix of walking distances between all houses and candidate tent locations. Because the algorithms do not guarantee that a globally optimal subset is found, we ran the *p*-center, *p*-median, and *p*-probability algorithms over 1000 iterations and kept the best solution for each. We assessed optimized placements by examining the distribution of walking distances between houses and their nearest vaccination point, the distribution of vaccine tent catchment sizes, the geospatial distribution of estimated vaccination probabilities, and the estimated overall vaccination coverage.

### Comparison of spatial evenness

We compared the spatial evenness of expected vaccination coverage achieved by sites used in the 2016 MDVC to those placed by our optimization algorithms by calculating the index of dissimilarity (D) of expected vaccination status^46^. The D index is a measure of how two mutually exclusive groups (in our case, households that do or do not participate in the vaccination campaign) are distributed among geographical units (in our case, city blocks); a value of 0 represents complete spatial evenness and deviations above 0 represents increasing spatial unevenness. It can be easily interpreted as the proportion of members of one group who would have to move out of areas in which they are overrepresented to areas in which they are underrepresented to produce perfect spatial evenness^46^.

D can be calculated as follows:

$$D = \frac{1}{2}\mathop \sum \limits_{i = 1}^{I} \left| {\frac{{n_{iv} }}{{N_{v} }} - \frac{{n_{iu} }}{{N_{u} }}} \right|$$

where *i* indexes city blocks; *I* represents the entire set of city blocks in the study area; *v* and *u* subscripts represent households that did and did not participate in the vaccination campaign, respectively; *n* represents the expected number of households that did (or did not) participate in the campaign and reside in block *i*; and *N* represents the total population of households that did (or did not) participate in the campaign.

### Computation and visualization

All analyses were performed in R^47^. We used the *MASS*^48^ and *glm* packages^49^ to fit regressions; the *tbart* package to optimize tent locations^45^; and *ggplot2*^50^ and *ggmap*^51^ packages to create figures. Base maps for all maps come from *OpenStreetMaps*^52^. Our code used to perform analyses is publicly available on GitHub^53^. https://github.com/RabiesLabPeru/RabiesMDVCOptimization.

## Results

### Regression construction of participation probability

**Figure 2:**
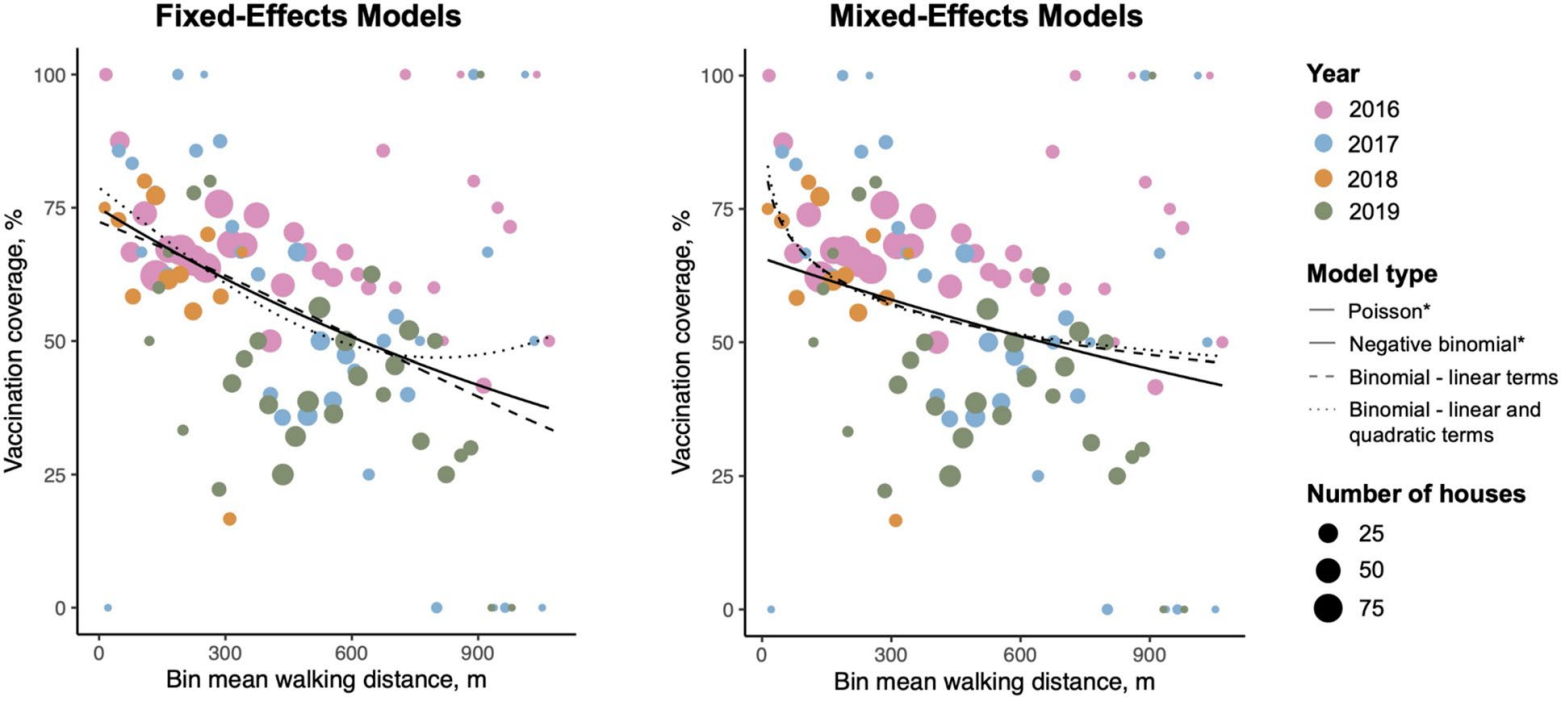
Regression models of the effect of distance on vaccination coverage. Regression curves are shown for the fixed-effects (left) and mixed-effects (right) models used to estimate the relationship between walking distance to the closest vaccination site and MDVC participation probability. Historical vaccination coverage data (colored dots) are visualized using 30 m binned distances, where dots are colored by year, scaled by the number of houses per bin, and plotted as the mean distance for all houses within a bin vs. the proportion of houses that participated in the MDVC for that bin. *Poisson and negative binomial regression curves are shown with a single line because coefficients were nearly identical for both fixed- and mixed-effects models.

Our regression analysis included 1855 household surveys from 2015 to 2019. We constructed and analyzed eight regression models to assess the relationship between travel distance to the closest vaccination tent and the probability of participation (Fig. 2). Based on the lowest AIC and best performance based on the smallest prediction error and root mean squared error (RMSE) via fivefold cross-validation, we selected mixed-effects Poisson regression as the best fit (Table 1). The results of the likelihood ratio test comparing the mixed-effects Poisson model to the mixed-effects negative binomial model was highly insignificant (χ^2^ = 0.0018, df = 3, *p* = 0.966), which further supports our selection of the mixed-effects Poisson model.

### Optimal placement of tents

**Figure 3:**
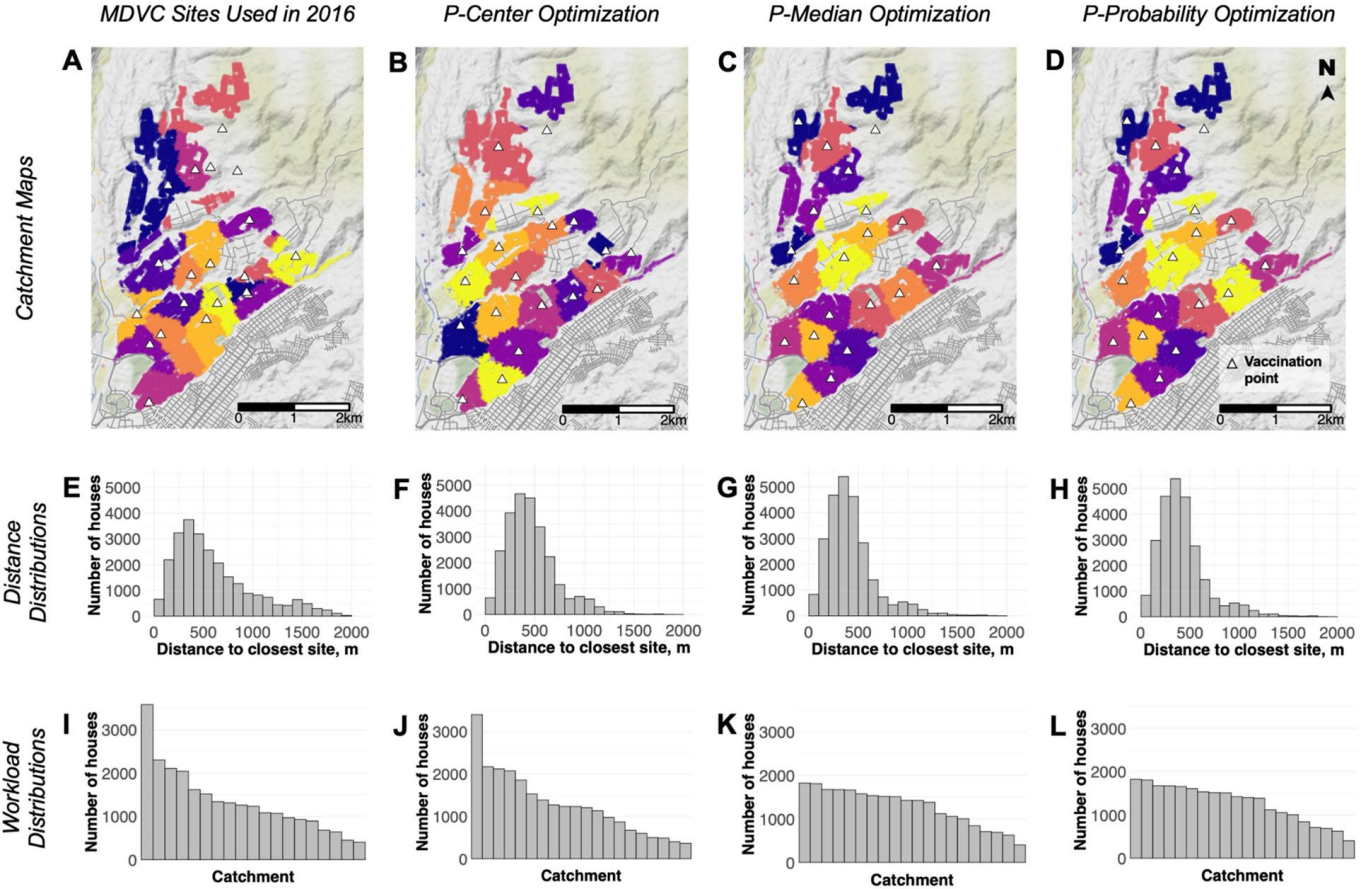
Vaccination point selection method comparison. Vaccination tent locations (white triangles) and subsequent catchment areas (different colored regions) are mapped based on different tent selection algorithms: **A** convenience, **B** *p*-center: minimized maximal walking distance, **C** *p*-median: minimized mean walking distance, and **D** *p*-probability: maximized participation probability. The middle-row histograms depict the distribution of distance to the closest vaccination point for the **E** convenience, **F** *p*-center **G** *p*-median, and **H** *p*-probability algorithms. The bottom-row histograms depict the corresponding workload distributions for the tents selected under the different algorithms **I**–**L**. The maps in panels A-D were created using R package *ggmap* version 4.0.0 (https://cran.r-project.org/web/packages/ggmap/).

Out of the 70 potential tent locations, the 20 most optimal tent locations were selected using four different methods: conventional placement, *p*-center optimization (minimized maximal walking distance), *p*-median optimization (minimized average walking distance), and *p*-probability optimization (maximized MDVC participation probability) (Fig. 3). Catchments are defined as the set of houses around a given vaccination tent for which that tent is the closest via walking distance. The selected tent locations and respective catchment areas for each method are displayed in Fig. 3A-D. The sites selected by the *p*-median and *p*-probability algorithms were very similar, differing by only one tent location in the east (3C-D). We compared the shortest walking distance distributions for the different sets of selected tent locations (Fig. 3E-G). The ideal distance distribution would be shifted towards zero as much as possible to minimize the distance traveled per household in the study area. The distributions of the *p*-median- and *p*-probability-optimized sites were shifted towards zero the most, as one would expect because these methods minimize overall walking distance (*p-*median) and maximize MDVC participation probability (*p*-probability), which decreases as a function of walking distance. We also assessed the workload for each tent location based on the number of houses in the catchment to determine whether selected sets resulted in uneven workloads across sites such that certain sites may be overloaded (Fig. 3I-L). An ideal distribution of workload would look uniform with the work being spread evenly across all catchments. Again, the *p*-median and *p*-probability algorithms performed the best (Fig. 3K-L).

**Figure 4:**
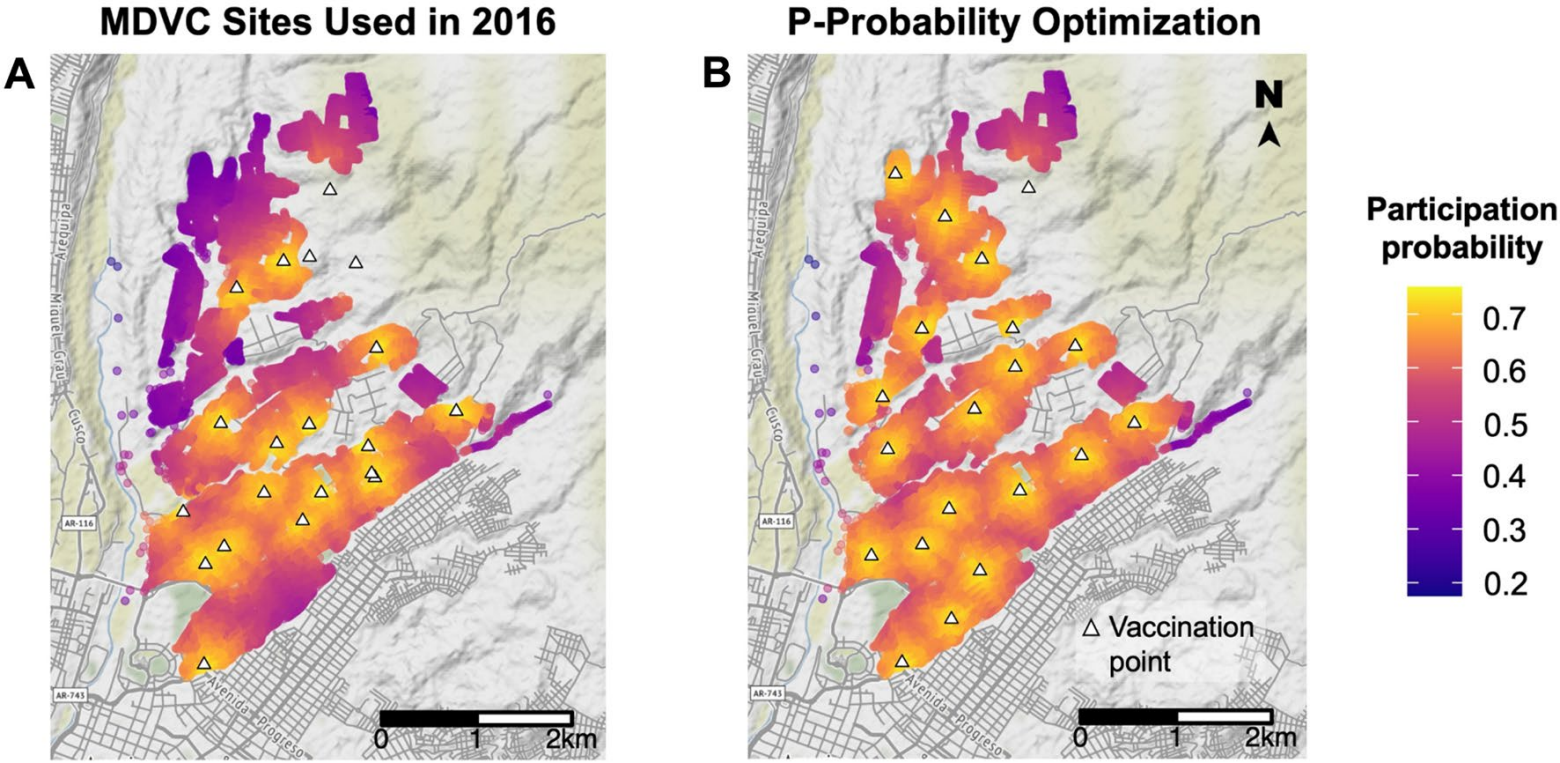
Predicted vaccination campaign participation. Panel **A** shows tent locations (white triangles) used in the 2016 MDVC, while panel **B** shows the optimized placement of tents obtained using the *p*-probability method. Houses (colored dots) are shaded according to their probability of participating in the MDVC, which was determined using our mixed-effects Poisson regression function with the random-effects coefficient for 2016 that related participation probability to distance to the nearest vaccination tent. These maps were created using R package *ggmap* version 4.0.0 (https://cran.r-project.org/web/packages/ggmap/).

The *p*-median and *p*-probability algorithms did better at both minimizing overall walking distance and evening out workload across vaccination tents. To assess the possible effects of these optimization methods on overall vaccination coverage, we applied the mixed-effects Poisson participation probability regression function to compare predicted participation in the vaccination campaign for the actual tent locations used in the 2016 campaign, which were selected based on convenience, the *p*-median-optimized locations, which were selected by minimizing the average walking distance to vaccination tents, and the *p*-probability-optimized locations, which were selected by maximizing expected participation in the vaccination campaign. The estimated vaccination coverage that would have been achieved using either the *p*-median- or *p*-probability-optimized sites was 63.2%, which represents a 7% increase in coverage compared to coverage estimated using actual 2016 MDVC locations (59.1%). The expected participation of households in the study area is mapped for actual 2016 MDVC locations versus *p*-probability-optimized sites in Fig. 4, and maps of expected participation using all site-selection methods are shown in Supp. Fig. 1.

### Comparison of spatial evenness

In addition to increasing overall vaccination coverage, spatially-optimized sites are expected to improve the spatial evenness of coverage across the study area, resulting in fewer undervaccinated pockets (Fig. 4, Supp. Fig. 1). We compared the spatial evenness between sites used in the 2016 MDVC and those placed by our optimization algorithms by calculating the index of dissimilarity (D) of expected vaccination status. The values of D for these sites are given in Table 2.

**Table 2:** Comparison of spatial evenness between current practice vaccination sites and vaccination sites optimized with different algorithms.

Sites used in 2016	*p*-center optimization	*p*-median optimization	*p*-probability optimization
0.157	0.109	0.105	0.105

Compared to sites used in 2016, all the optimization algorithms considered produced a smaller D value, indicating that these algorithms improved the spatial evenness of expected vaccination coverage. The *p*-median and *p*-probability algorithms, which performed best in terms of optimizing total vaccination coverage, also improved spatial evenness the most. The 33% reduction of D achieved by the *p*-median and *p*-probability algorithms can be interpreted as follows: compared to the actual vaccination sites used in 2016, if sites placed by these algorithms were used, one-third fewer participant and non-participant households would have to move to produce an even spatial distribution of vaccination coverage.

## Discussion

We combine techniques used in facility location problems, combinatorial optimization, and statistical modeling to optimally place fixed-location vaccination points for the annual mass dog rabies vaccination campaigns in Arequipa, Peru. In line with previous studies examining barriers to vaccination^13,18,54–56^, we found a significant negative association between walking distance from a vaccination location and household participation in the vaccination campaign. In order to maximize the coverage obtained from a fixed number of vaccination points in the city, we optimized the locations of the vaccination points by either minimizing walking distance to the vaccination tents (*p*-median and *p*-center) or maximizing overall participation probability (*p*-probability) The *p*-median and *p*-probability methods performed best at increasing estimated vaccination coverage and evening workload across vaccination sites. In addition, all optimization methods noticeably improved spatial evenness in expected vaccination coverage compared to conventional placement.

Compared to historical data with tents placed using conventional methods, we predict based on our mixed-effects Poisson regression function that coverage would increase by 7% if either the *p*-probability or *p*-median optimization algorithm was applied. The algorithms we developed use a data-driven approach to combat an ongoing dog rabies epidemic by optimally using limited resources to maximize vaccination coverage. The main effects we expect from spatially optimizing vaccination sites would be increased overall vaccination coverage and increased spatial evenness of coverage. The Pan American Health Organization recommends that 80% of dogs get vaccinated annually to reach population immunity levels effective to eliminate canine rabies from a region^57^. Our optimization algorithm helps us get closer to this goal. The World Health Organization has set a goal of zero human deaths from rabies by 2030^58^. The most effective way to prevent dog-mediated human rabies is to control rabies in the canine population through mass dog vaccination^59^; however, this requires sustained yearly vaccination campaigns requiring the mobilization of health officials and dog owners^6^.

Facility location optimization can help determine how to best use limited resources; this is especially important for a neglected disease that suffers globally from underfunded control programs^60^. Canine rabies control programs are underfunded globally and this is certainly the case in Arequipa, Peru. Control programs worldwide are under additional strain as the COVID-19 pandemic has diverted funds and resources away from other public health initiatives^11^. The algorithm we present here can help to best locate vaccination facilities. Hence, this application of the facility location problem can be used beyond rabies. Any mass vaccination campaign that sets up fixed-location points to which people travel for vaccinations^13,61,62^ could be enriched by these algorithms as long as travel distance is a barrier to access.

In our study of MDVCs in Arequipa, Peru, the selection of vaccination sites obtained by the *p*-probability and *p*-median algorithms were very similar, differing by only one site and resulting in near-equal estimates of expected vaccination coverage. However, applying these algorithms in other contexts with different spatial distributions of residences and candidate facility sites or with a different dependence between participation probability and travel distance is likely to yield different results. For instance, in Tanzania, it was found that vaccination coverage fell below a 70% target threshold only at distances greater than 5 km from the vaccination sites^63^. While the *p*-probability algorithm is designed to maximize MDVC participation and hence overall vaccination coverage, it is important to balance consideration of overall coverage with spatial evenness of coverage as undervaccinated pockets can sustain an epidemic even when herd-immunity thresholds have been reached^32,35^. By minimizing the maximum distance any household has to travel to a facility, the *p*-center algorithm may be particularly well-suited for promoting spatial equity and has been utilized by others to increase equity of access to healthcare and other services^64,65^. Workload distribution across facility sites is another, less-often-discussed factor that can influence the success of a vaccination campaign, as a high workload at a site may result in long waiting lines that can compromise delivery. Here, we found that the *p*-probability and *p*-median algorithms were the most effective in improving overall vaccination coverage, spatial equity, and workload distributions. However, the optimal algorithm may vary in different contexts, depending on the setting and the relative importance assigned to these different factors.

While fixed-point vaccination locations are more cost-effective than other methods such as door-to-door vaccination^59^, many MDVC’s deploy both fixed-point and door-to-door vaccination strategies to increase coverage^13,66,67^. For example, vaccination teams may operate out of fixed-point vaccination sites for the initial 2 weeks of a campaign, and then vaccinate door-to-door the following 2 weeks to target areas with low turnout at the fixed sites. However, deciding which areas to target with door-to-door campaigns is a challenge. Our approach, which utilizes a regression function to estimate fixed-point MDVC participation probability based on a household’s walking distance to the nearest vaccination site, can be used to identify high-risk areas (those most likely to experience under-vaccination) that should be visited by door-to-door vaccination teams (Fig. 4). Such an approach can be applied broadly to benefit any vaccination program that combines fixed-point and door-to-door strategies, which include COVID-19 vaccination programs deployed in the US^68–70^, Canada^71^, India^72^, and Malawi^73^.

We developed a computational solution to optimally place vaccination points by maximizing the overall probability of participation based on walking distance. Based on our Poisson regression, we predict that this will increase overall vaccination coverage and vaccination coverage evenness in our study area. Our future directions include validating this method via a field trial. One limitation of our study is that the approach has only been tested in silico. Our future directions include validating this method via a field trial. Another limitation is that we looked solely at walking distance from a vaccination point to determine the probability of MDVC participation, although many factors influence participation^13^. Considering these factors would strengthen strategies to increase vaccination coverage. Additionally, our algorithm does not account for workload variation due to different household densities, which could create waiting lines at the vaccination sites and increase abandonment rates. Finally, while our approach does not require additional costs for vaccinators and field workers, it will require a modest budget for computational power and programming time. On the other hand, some important strengths of our work are that the approach is data-driven and does not require a lot of computing power or time to implement. Moreover, it is a flexible approach that can be applied to any environment where the locations of houses and potential vaccination sites are known.

Vaccine equity, fair access to vaccines for everyone, is essential for eliminating the rabies virus, which can circulate at very low levels within dog populations^32^ and persist in localized pockets of under-vaccinated dogs^35^. Achieving high vaccination coverage alone is insufficient; rather, it is crucial to ensure equitable distribution of this coverage across the entire population^34^. By optimizing the location of vaccination points, access to the vaccine is balanced across the study area. When locations are selected based on convenience, neighborhoods that are more isolated are disproportionately disenfranchised from access to rabies vaccinations.

Spatial access has affected vaccine equity for other programs. For instance, the geographic distribution of COVID-19 vaccine delivery locations had implications for vaccine equity^56^, with a higher risk of COVID-19 disease and severity with longer travel times to vaccination sites. Spatial accessibility to COVID-19 vaccination services was better in urban areas compared to rural areas^74^, and within urban areas, spatial access to COVID-19 vaccination centers was worse in the periphery and poorer areas of cities^75^. Using a data-driven approach to optimally place vaccination sites could increase vaccine coverage and vaccine equity, and increase the chances of control or elimination.

### Supplementary Information

Supplementary Information.

## Data Availability

The datasets generated during and/or analyzed during the current study are available from the corresponding author on reasonable request.
