# Supplementary material for: Optimizing the location of vaccination sites to stop a zoonotic epidemic
Source: Sci Rep. 2024 Jul 10;14:15910. doi: 10.1038/s41598-024-66674-x (PMC11237137; doi:10.1038/s41598-024-66674-x)

**Optimizing the location of vaccination sites to stop a zoonotic epidemic**

**Supplementary Figure 1. Predicted vaccination campaign participation for sites used in 2016 (A) and sites optimized by the *p*-center (B), *p*-median (C), and *p*-probability (D) algorithms.**Vaccination sites are depicted as white triangles, and houses are depicted as colored dots shaded according to their probability of participating in the MDVC, which was determined using the mixed-effects Poisson regression function with the random-effects coefficient for 2016 that related participation probability to distance to the nearest vaccination site. The estimated vaccination coverages are given by the bold numbers in the top right of each map. All maps were created using R package *ggmap* version 4.0.0 (<https://cran.r-project.org/web/packages/ggmap/>).


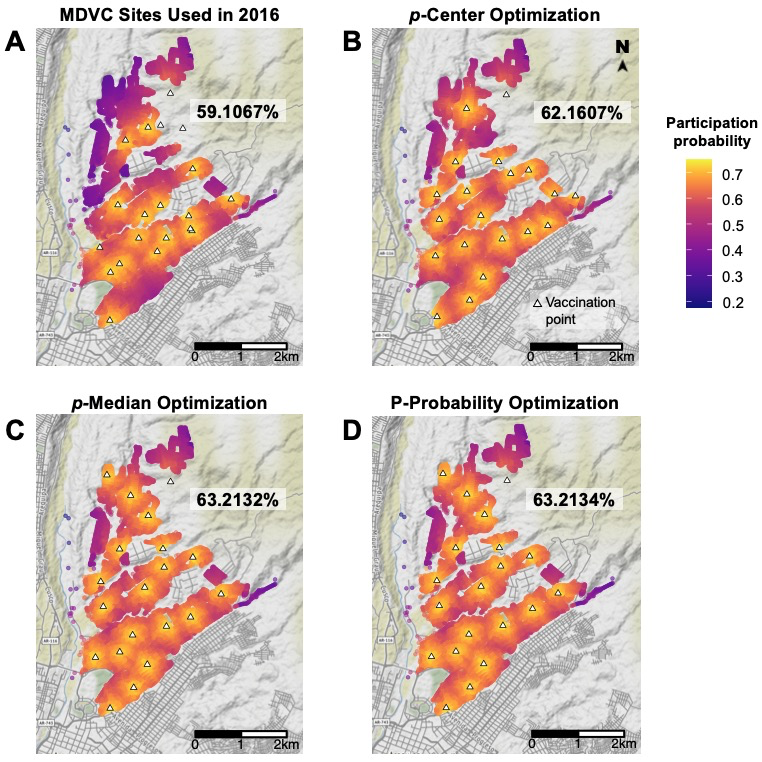

Supplement: Supplementary file 1 — Supplementary Information. [file 41598_2024_66674_MOESM1_ESM.docx]
